# Deep Phenotyping and Molecular Elucidation of a New Syndrome: Ectodermal Dysplasia Caused by *IRF6* Variants

**DOI:** 10.1111/exd.70331

**Published:** 2026-07-22

**Authors:** Holm Schneider, Smail Hadj‐Rabia, Carola Berking, Matthias Weider, Julie Steffann, Ralf J. Rieker, Lina Gölz, Nicolai Peschel

**Affiliations:** ^1^ Center for Ectodermal Dysplasias University Hospital Erlangen Erlangen Germany; ^2^ Department of Pediatrics Friedrich‐Alexander‐Universität Erlangen‐Nürnberg Erlangen Germany; ^3^ Department of Dermatology, Reference Centre for Genodermatoses and Rare Skin Diseases (FIMARAD) Hopital Necker‐Enfants Malades, INSERM 1163 Paris France; ^4^ Department of Dermatology and Venereology Friedrich‐Alexander‐Universität Erlangen‐Nürnberg Erlangen Germany; ^5^ Department of Orthodontics and Orofacial Orthopedics Friedrich‐Alexander‐Universität Erlangen‐Nürnberg Erlangen Germany; ^6^ Department of Molecular Genetics Hopital Necker‐Enfants Malades, INSERM 1163, Institut Imagine Paris France; ^7^ Department of Pathology Friedrich‐Alexander‐Universität Erlangen‐Nürnberg Erlangen Germany

**Keywords:** anhidrosis, ectodermal dysplasia, hearing loss, interferon regulatory factor 6, natal teeth

## Abstract

The diagnosis of an ectodermal dysplasia (ED) is often made by dermatologists. Some of the more than 50 distinct ectodermal dysplasias, however, are still largely unknown and their pathogenesis is poorly understood. Since we recently discovered that variants of the Interferon Regulatory Factor 6 (IRF6) gene *IRF6* may cause ED, we have further investigated the link between this gene and maldevelopment of tissues originating from the embryonic ectoderm. Disease characterization in two previously reported subjects and one newly identified patient (mosaic status) included systematic clinical and genetic evaluation, assessment of all available patient records and dental radiographs, hearing tests and immunostaining of skin samples. Structural models of native and mutant IRF6 were analysed to elucidate the pathogenesis. IRF6‐associated ED, a combination of natal teeth (irregularly), absent sweat glands, hidradenitis suppurativa‐like symptoms, onychodysplasia, agenesis of numerous deciduous and permanent teeth, and sensorineural hearing impairment, was linked to amino acid substitutions in a region between the DNA‐binding domain and the protein‐binding domain of IRF6, which has not yet been assigned a function. Genotype–phenotype correlations from a total of five patients and immunohistochemical data support the assumption that IRF6‐associated ED is based on a gain‐of‐function mechanism. Thus, *IRF6* variants are not only the cause of Van der Woude syndrome and popliteal pterygium syndrome, two diseases with cleft lip/palate as common features, but are most likely responsible also for a new, challenging syndrome without orofacial clefting. Our results facilitate accurate and early diagnosis in affected individuals and may pave the way for specific treatment.

## 1 Introduction

Ectodermal dysplasias are rare genetic disorders of the development and/or homeostasis of two or more ectodermal derivatives, including hair, teeth, nails and certain glands [1]. The most common one, X‐linked hypohidrotic ectodermal dysplasia (XLHED), is characterized by hypodontia, sparse, light‐coloured hair, hypohidrosis resulting from the lack of sweat glands, and structural and/or functional deficits of sebaceous, meibomian, salivary and mammary glands [2]. Some ectodermal dysplasias lead to severe congenital malformations of craniofacial structures and digits [1]. For example, pathogenic variants of the transcription factor gene *TP63* cause ectrodactyly‐ectodermal dysplasia‐clefting (EEC; MIM 129900) [3] or ankyloblepharon‐ectodermal dysplasia‐clefting (AEC; MIM 106260) [4] syndromes. Both are characterized by cleft lip and/or palate (CL/CP), a feature which can also be found in patients with heterozygous pathogenic changes in *IRF6*, the gene encoding Interferon Regulatory Factor 6 (IRF6) [5]. Pathogenic *IRF6* variants have usually been associated with Van der Woude syndrome 1 (VWS1; MIM 119300) and popliteal pterygium syndrome (PPS; MIM 119500) [5] that do not belong to the ectodermal dysplasias. Patients with VWS1 have CL/CP with lower lip pits, whereas PPS additionally includes webbing of the skin, syndactyly and other anomalies [5]. In 2025, we reported briefly on a new type of ectodermal dysplasia due to a heterozygous missense mutation in exon 6 of *IRF6* [6], located between the regions that code for the DNA‐binding domain and the protein‐binding domain of IRF6 [7, 8]. The genomic region comprising exons 5 and 6 of *IRF6* has not yet been assigned a function. Prior to our report, missense mutations had almost exclusively been detected in exons 3, 4, 7, 8 and 9. The two unrelated patients whom we described [6] both had neither CL/CP nor lip pits but suffered from anhidrosis and had each been born with numerous teeth. Thus, *IRF6* was linked to another rare disease entity, which expands the current classification of ectodermal dysplasias [9]. We initially referred to this new disease as Ectodermal Dysplasia with Natal Teeth (EDNAT) and assumed that more patients with a similar phenotype will be identified and diagnosed to carry *IRF6* variants.

Here we present complete phenotypic data of these two individuals and another patient with an amino acid substitution in IRF6 just two amino acids away from the substitution reported previously.

## 2 Material and Methods

The study was approved by the ethics committee of Friedrich‐Alexander‐Universität Erlangen‐Nürnberg (reference number 559_20B). All participants provided written informed consent.

DNA samples isolated from EDTA blood of the patients and their family members were investigated by whole exome, whole genome or Sanger sequencing. High‐throughput DNA sequencing was performed using an Illumina Hiseq sequencer NovaSeq6000 (2 × 101 bp), followed by bioinformatic analysis on the Illumina Dragen platform (patient 1), which identified a heterozygous *IRF6* variant, c.653 T>A (A: 56 | T: 62; average reading depth of 130×). To confirm its de novo status, the entire coding region of *IRF6* from the patient's parents was analysed by Sanger sequencing. The ACMG‐based classification suggested a variant of unknown significance, but we consider a confirmed de novo variant as likely pathogenic. Investigation of asymptomatic family members focused on exon 6 of *IRF6*.

In order to establish a molecular genetic diagnosis for patient 2, Sanger sequencing of *IRF6* was carried out after no pathogenic variants had been identified in the previous sequencing of *EDA*, *EDAR*, *EDARADD* and *TP63*. Patient 3 and her parents were investigated by trio whole genome sequencing without orthogonal confirmation analysis. Phenotyping included systematic clinical examinations and photo documentation, assessment of the dental status and/or existing panoramic X‐ray images, confocal laser scanning microscopy of the palms, and standardized measurement of sweat production within 30 min following stimulation with pilocarpine in a small area of the forearm (Wescor 3700, Wescor Inc.) as described previously [10].

Skin biopsy specimens were obtained from the upper limbs of two patients with local anaesthesia and stained with standard techniques using a goat polyclonal anti‐IRF6 antibody (Novus Biological, NBP1‐51911, dilution 1:100), DAPI (4′,6‐diamidino‐2‐phenylindole, dilution 1:1000), polyclonal donkey anti‐goat IgG (Invitrogen catalogue # A‐11058), dilution 1:200, a monoclonal antibody against keratin 17 (Epitomics, Cytokeratin 17 EP98, dilution 1:100), 3,3′‐diaminobenzidine and counterstaining with haematoxylin.

Models of the IRF6 structure based on the full‐length amino acid sequence of monomeric human IRF6 or the same sequence with an Ile218Asn or Ser220Pro exchange [11] were generated with AlphaFold3. Molecular analysis was performed using UCSF ChimeraX, developed by the Resource for Biocomputing, Visualization and Informatics at the University of California, San Francisco [12].

## 3 Results

**FIGURE 1:**
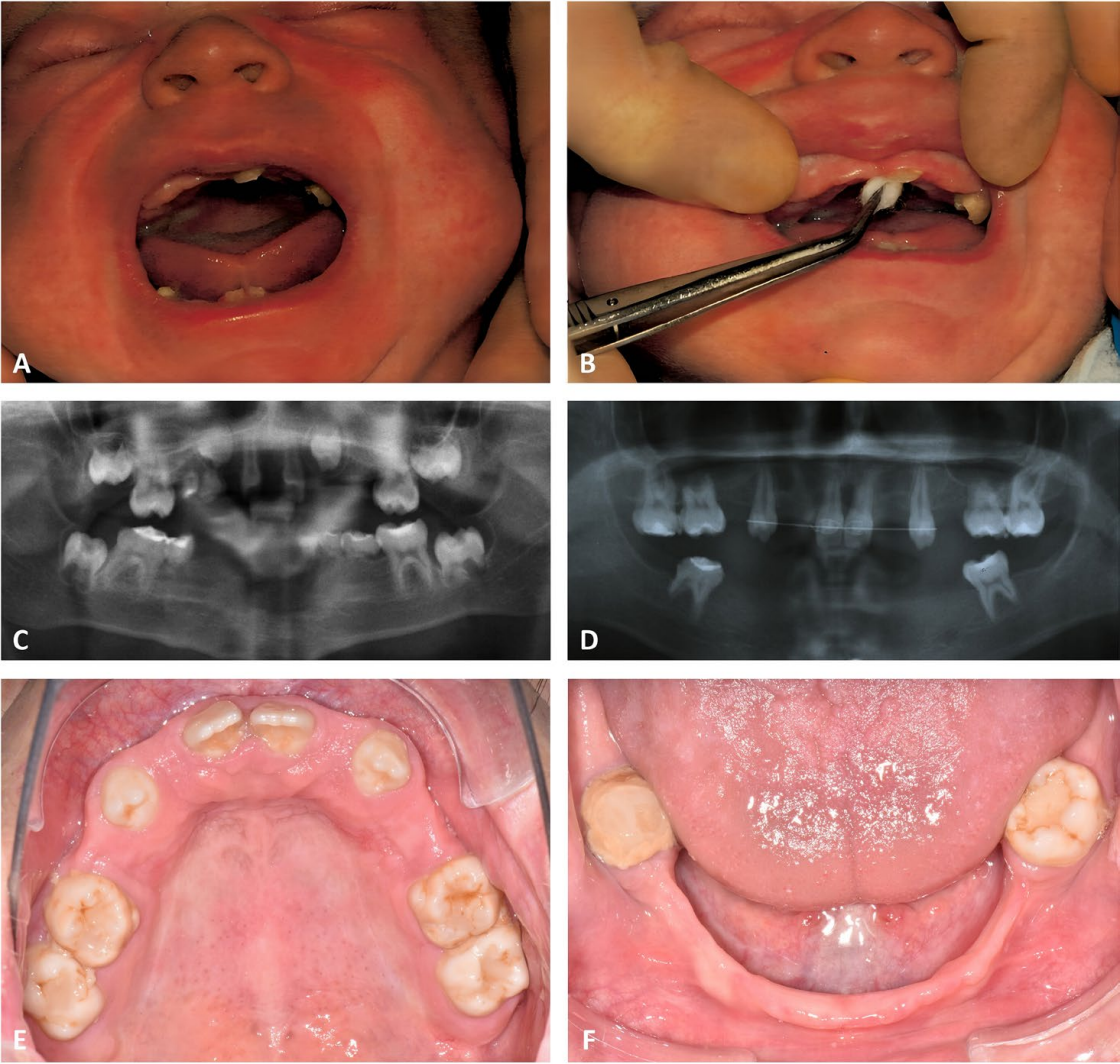
Dentition of patient 1. Facial photographs of the two‐week‐old infant showing five of six natal teeth (A), the different shape of which is presented (B). Panoramic dental X‐rays at the age of 6.5 years (C) and 11 years (D). Dental status at the age of 17 years (E, F). Note the highly atrophic alveolar crest in the lower jaw (F). The patient consented to publication of these images.

Patient 1 (P1) and patient 2 (P2), 17 and 14 years old, respectively, are unrelated male teenagers who had become noticeable already on their first day of life due to natal teeth (Figure 1A,B). These weakly anchored teeth were either lost spontaneously or extracted in early infancy without problems. P1 then developed 7 deciduous teeth with some delay (Figure 1C); 12 permanent teeth erupted, two of which had to be removed due to repeated dental fistula or cysts, so that only 10 teeth remained (Figure 1D). Most of them exhibited enamel defects and enamel hypomineralization (Figure 1E,F), which resulted in the necessity for multiple fillings due to hypersensitivity and an elevated risk of caries. As a consequence of oligodontia, particularly in the lower jaw, the alveolar crest was highly atrophic (Figure 1F), and substantial bone augmentation prior to future treatment with osseointegrated dental implants will be needed. P2 developed only 9 permanent teeth; two molars had to be extracted as they were displaced and impacted. Both patients experienced episodes of overheating and difficulty regulating the body temperature since early infancy and suffered from recurrent skin abscesses, especially in the genital/gluteal area, behind the ears, under the armpits and in the mouth. In general, the skin was very dry, with plantar hyperkeratosis and blistering on the backs of the toes during the winter months. Investigations of the capability to perspire revealed anhidrosis, and no sweat glands were detectable on the palms by confocal laser scanning microscopy. Some toe‐ and fingernails were dysplastic, thickened or deformed with distal onycholysis. Increased production of thick mucus in the nose and upper throat was reported, and the ears were partially filled with dry cerumen. Both subjects had a bilateral sensorineural hearing impairment since infancy, currently being treated with hearing aids. A submucous cleft palate was excluded in each case. P1 also showed a tendency to allergies, which manifested in several anaphylactic reactions to milk protein (among other antigens).

Molecular genetic analysis of blood cells from these unrelated individuals revealed the same heterozygous substitution, c.653 T>A, chr1‐209 965 628 A>T, in exon 6 of the *IRF6* gene, which led to an Ile218Asn exchange in the encoded protein predicted to be deleterious. It was categorized as *de novo*, because the parents' DNA showed no alteration at this position.

In patient 3 (P3), a 22‐year‐old woman referred for anhidrosis, dry skin and intolerance to environmental temperatures above 22°C, diffuse xerosis and dilatation of the follicular ostia were noted. The woman had experienced several episodes of skin abscesses mimicking hidradenitis suppurativa. Bilateral lesions in the groins, axillae and intergluteal fold had been treated with antibiotics and surgery. Scalp hair, eyebrows and eyelashes were developed normally. This patient also had pincer nails on both big toes. She showed no syndactyly and normal breasts and nipples. A bilateral acquired sensorineural hearing loss was diagnosed (profile curve resembling presbyacusis with tonal audiometry thresholds of 1000–2000 Hz at 40–50 dB). The ear canals were large with a healthy eardrum and a tendency to retraction (discrete deformation of the external ear canal) and atelectasis (confirmed by a computer tomography scan of the petrous bones). Natal teeth were not remembered. Lips and palate displayed no abnormality, but episodes of epistaxis and crusted rhinitis were reported and the woman complained about insufficient salivation. Ocular examination due to gritty eyes revealed bilateral superficial punctate keratitis. The tear film break‐up time was reduced (4–5 s).

As this subject had no family history of ectodermal dysplasia and non‐consanguineous healthy parents, trio whole genome sequencing with a mean reading depth of 48× was performed and identified a heterozygous de novo *IRF6* variant, c.658 T>C (p.Ser220Pro), with a variant allele frequency of 30.5% (T: 42 | C: 18) indicating mosaicism. In silico analyses support its pathogenicity (MobiDetails, CADD25). Other disease‐causing gene variants were excluded. Considering that the patient presented with anhidrosis, hypolacrimation and hyposalivation, symptoms that do not match the typical clinical picture associated with *IRF6* mutations, the variant was ultimately classified as of unknown significance.

The presumably disease‐causing variants in subjects P1, P2 and P3 affect the same amino acid residues as in two additional patients (P4 and P5) recently described by a research group from China [13]. Table 1 summarizes the various symptoms in all known patients with IRF6‐associated ED in comparison with the known symptoms of VWS1, PPS and p63‐related disorders. A combination of oligodontia, hypo‐ or anhidrosis, hidradenitis suppurativa‐like skin abscesses, poikiloderma, onychodysplasia and bilateral hearing impairment is considered to be syndrome‐defining, while natal teeth and sparse hair were observed irregularly or may be variable. A significant proportion of the clinical changes which are characteristic of VWS1, PPS, or the new disease entity also occur in patients with p63‐related diseases. Nevertheless, certain symptoms are specific to a particular disease. For instance, popliteal pterygia seem to be observed exclusively in PPS and ectrodactyly is a distinct feature of p63‐related disorders.

**TABLE 1:** Characteristics of IRF6‐associated ED, VWS1, PPS and p63‐related disorders.

Symptoms	P1	P2	P3	P4 (Zhang et al.)	P5 (Zhang et al.)	VWS1	PPS	p63‐related disorders
	IRF6 Ile218Asn		IRF6 Ser220Pro	IRF6 Ile218_Ser220delins	IRF6 Ile218Asn			
Lip pits	−	−	−	−	−	+	+	−
Cleft lip/palate	−	−	−	−	−	+	+	+
Natal teeth	+	+	−	−	−	−	−	−
Oligodontia	+	+	+	+	+	−	−	+
Hypo‐ or anhidrosis	+	+	+	+	+	−	−	+
Hidradenitis suppurativa‐like skin abscesses	+	+	+	+	+	−	−	−
Poikiloderma	+	+	+	+	+	−	−	−
Bilateral hearing impairment	+	+	+	+	+	−	−	+
Nail dysplasia	+	+	+	+	+	−	−	+
Sparse hair	−	−	−	+	+	−	−	+
Popliteal pterygia	−	−	−	−	−	−	+	−
Pyramidal skin on hallux	−	−	−	−	−	−	+	−
Ankyloblepharon	−	−	−	−	−	−	rare	+
Split hand/ft, syndactyly	−	−	−	−	−	−	rare	+
Hypoplastic nipples or breasts	−	−	−	−	−	−	−	+

**FIGURE 2:**
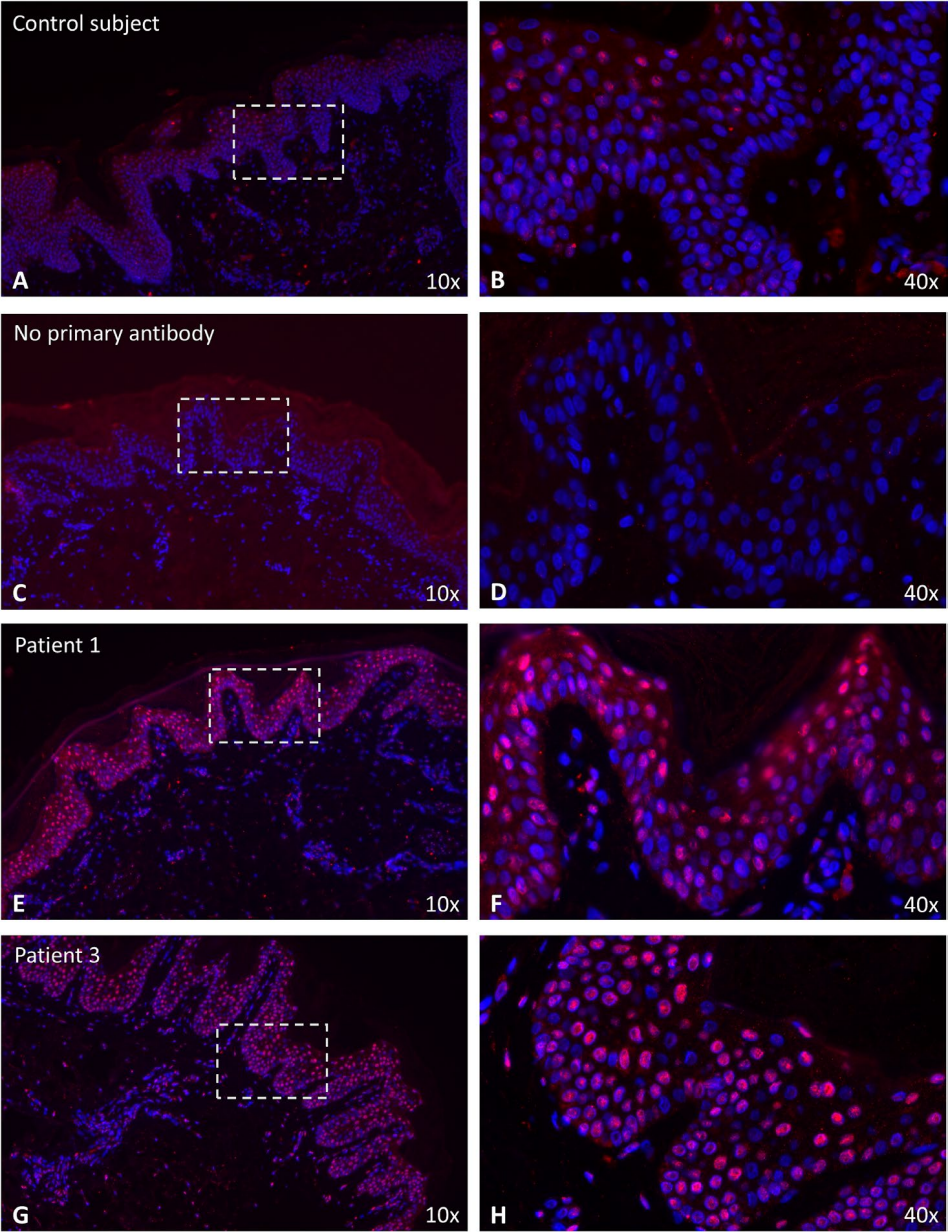
Immunohistochemical detection of IRF6 in skin sections. Normal adult skin from a healthy control subject was stained for IRF6 (A) and is shown together with a negative control staining (C) and corresponding close‐up of the epidermis (B and D, respectively). Skin samples from patient 1 (E, F) and patient 3 (G, H) were stained in the same manner. IRF6 is indicated by red colour. The sections were counterstained with DAPI to mark cell nuclei.

Histological analysis of skin samples from P1 and P3 showed focal subcorneal clefting and basal spongiosis. Immunofluorescence staining for IRF6 protein in healthy control skin revealed its expression especially in the epidermis (Figure 2A,B). IRF6 was not detected when no primary antibody was used (Figure 2C,D). The nuclei of keratinocytes from P1 showed stronger specific staining (Figure 2E,F) than control nuclei (Figure 2A,B), while an apparently more patchy nuclear staining was observed in the epidermis of P3 (Figure 2G,H), fitting with genetic mosaicism. Sweat glands were absent in both patients.

**FIGURE 3:**
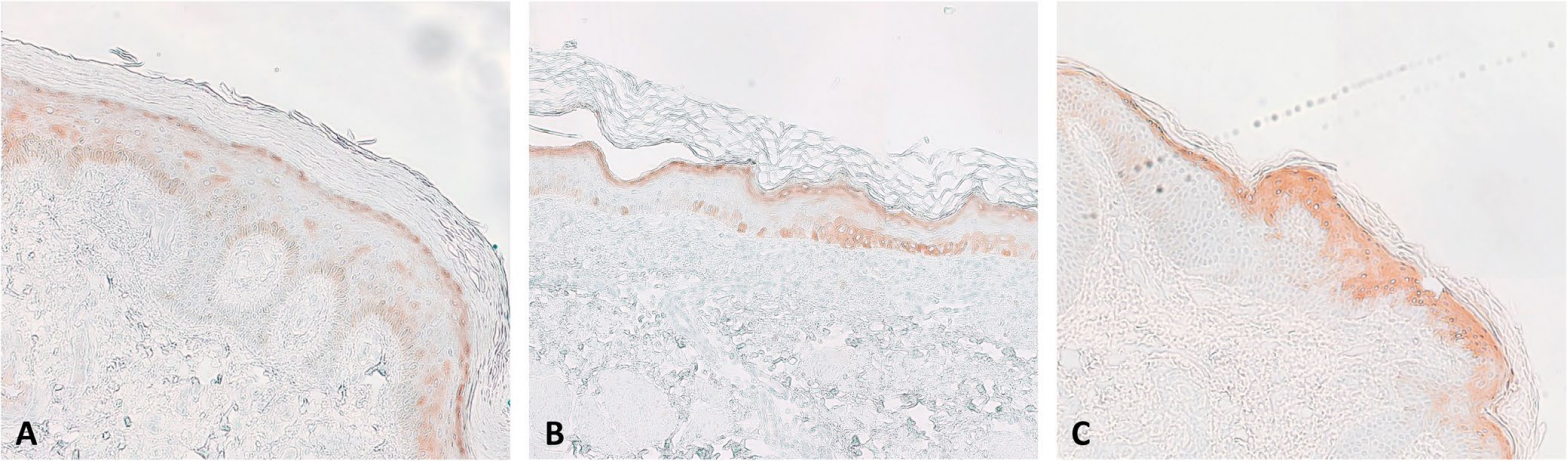
Immunostaining of skin sections for keratin 17. Skin biopsy specimens from a healthy control subject (A), patient 1 (B) and patient 3 (C) were stained with an antibody recognizing keratin 17, which is indicated by reddish colour. The sections were counterstained with haematoxylin.

As natal teeth have been a typical feature of Pachyonychia congenita 2 (MIM 167210) caused by pathogenic *KRT17* variants [14] and loss of IRF6 in the periderm can result in abnormal KRT17 expression [8], we also stained the patient's skin biopsy specimens for keratin 17 but did not observe any anomalies (Figure 3).

**FIGURE 4:**
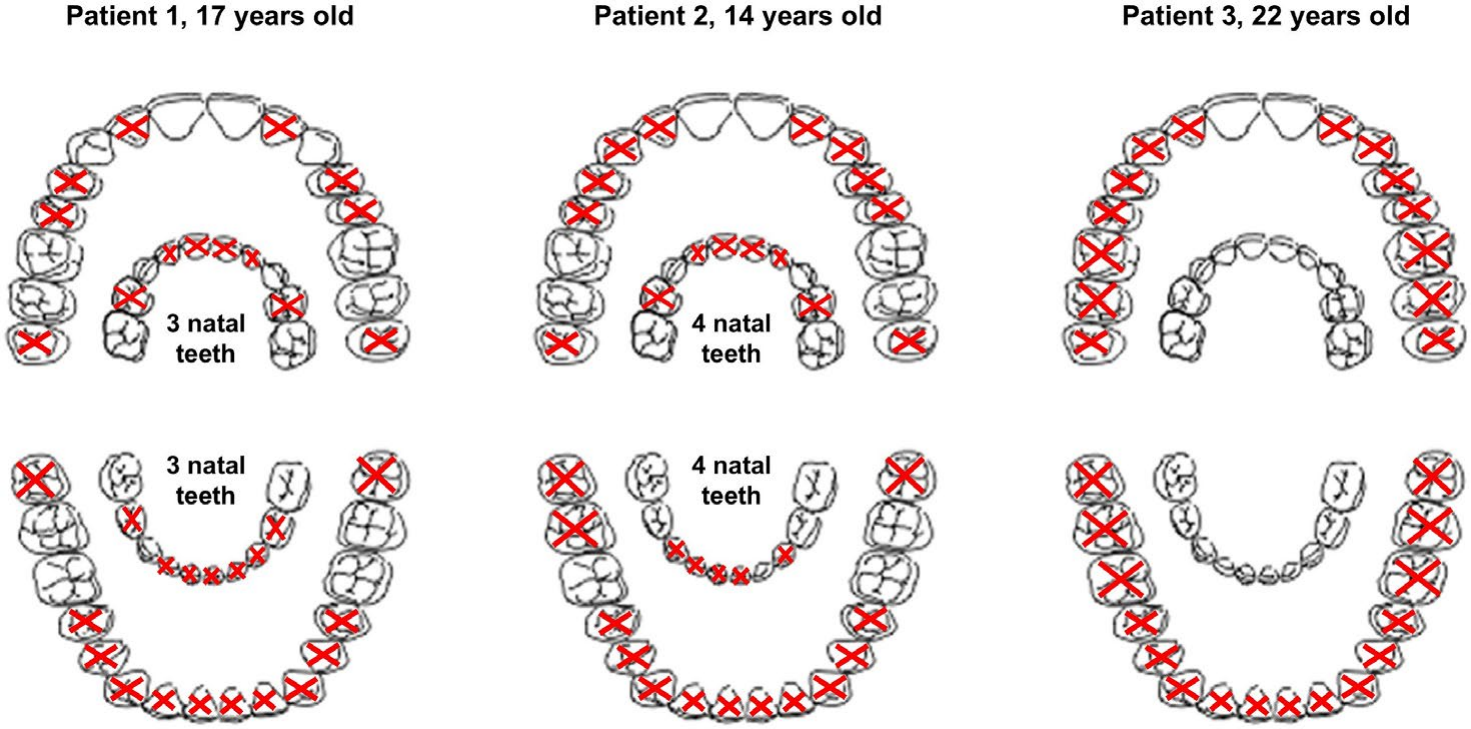
Pattern of tooth agenesis in IRF6‐associated ED. Tooth agenesis is marked by a red cross at the indicated position.

When comparing the dentition of P1, P2 and P3, a pattern of tooth agenesis was recognized. All three patients had permanent incisors in the upper jaw, but no incisors or canines in the mandible, and none of these unrelated individuals had any permanent premolars (Figure 4). Due to the lack of teeth in the mandible, a pronounced atrophy of the lower alveolar crest was evident. Interestingly, patient 3 presented with 16 persisting deciduous teeth and only two permanent teeth after delayed tooth eruption.

## 4 Discussion

**FIGURE 5:**
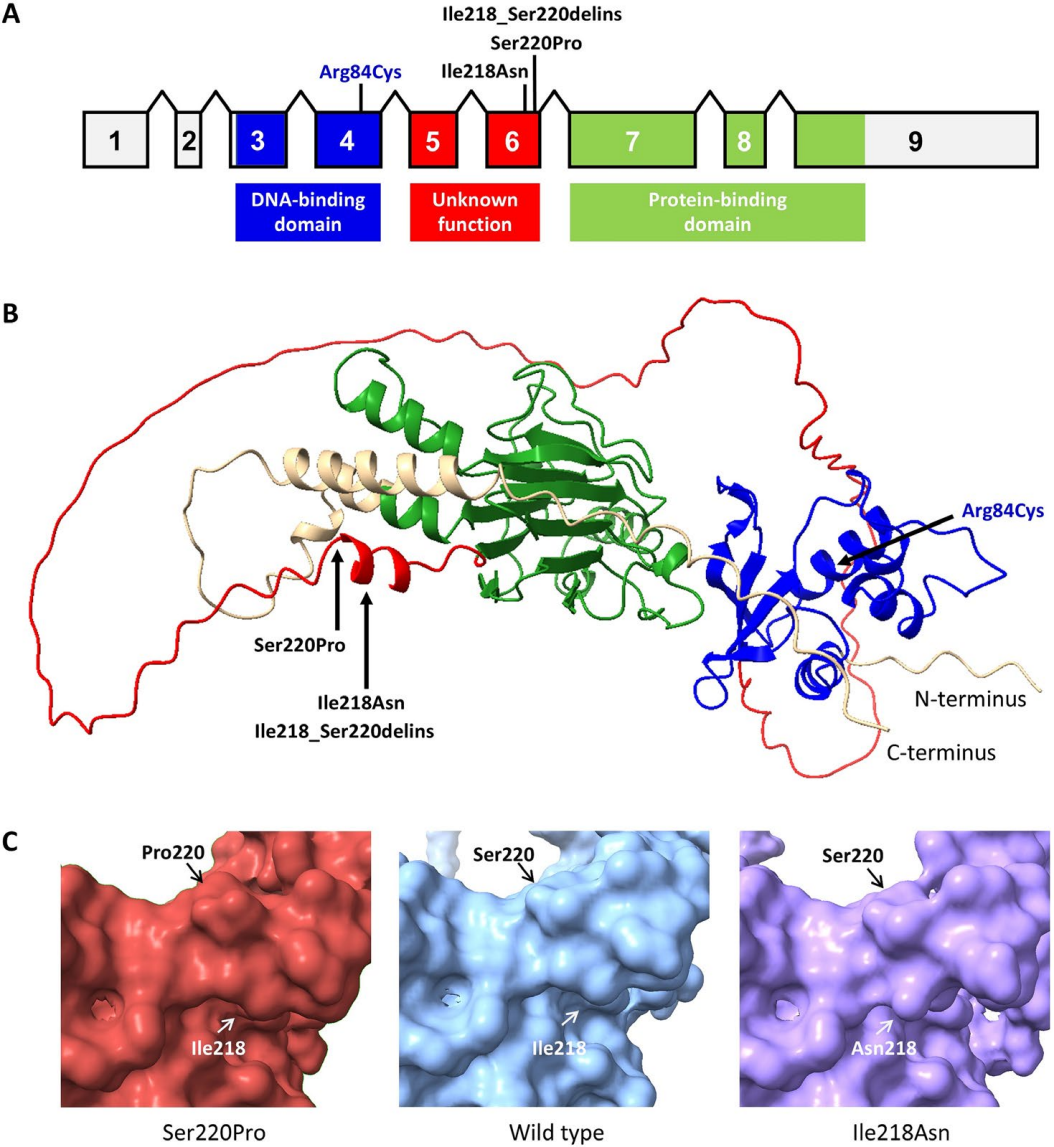
Pathogenic *IRF6* variants and their possible effects on the protein. (A) Scheme of the genomic structure of *IRF6*. Untranslated regions are represented by grey boxes, regions that encode specific domains of the protein are shown in blue (DNA‐binding domain, amino acids 13–113), red (unknown function) or green (protein‐binding domain, amino acids 226–394). The lines between the boxes indicate introns. PPS is associated with the mutation p.Arg84Cys in exon 4, while IRF6‐associated ED is caused by amino acid substitutions in exon 6. (B) Prediction of the IRF6 structure (AlphaFold 3). The regions of interest are indicated by the same colours as before. (C) A close‐up of the protein's surface in the area of the point mutations in exon 6 compared with the wild type.

Interferon Regulatory Factor 6 is a transcription factor with a highly conserved helix‐turn‐helix DNA‐binding domain and a Smad‐interferon regulatory factor‐binding domain [15]. Various mutations in the corresponding gene have been reported. So far, pathogenic *IRF6* variants have been associated only with isolated cleft lip or cleft palate (Orofacial Cleft 6; MIM 608864) [16], CL/CP with lip pits (VWS1) [5], and CL/CP combined with genital and skin abnormalities including webbing on the lower limbs (PPS) [5]. Our group was the first to describe an association of *IRF6* with ectodermal dysplasia (EDNAT) [6]. Why and how can *IRF6* variants lead to such a wide range of phenotypes as seen in VWS1, PPS or the new disease entity? The gene structure may give an initial answer. *IRF6* consists of 9 exons, of which only exons 3–9 are translated (Figure 5A). Remarkably, the distribution of missense mutations that lead to a particular clinical picture is not even across the gene [7, 8, 17]. While protein‐truncating variants and deletions are distributed randomly over all exons and mainly cause VWS1, missense mutations are mostly found in exons 3, 4, 7, 8 and 9. Pathogenic variants that lead to PPS almost exclusively affect exons 3 and 4 (Figure 5A) which carry the information for the DNA‐binding domain [8]. Exons 5 and 6 encode a less conserved proline‐rich region with unknown function. Unfortunately, the 3D structure of the protein does not allow any conclusions about the role of this region. Since no crystal structure of IRF6 has yet become available, we generated a structural model *in silico* using AlphaFold3 (Figure 5B,C). The per‐residue model confidence score [18] for the DNA‐ and protein‐binding domains was very high (mostly above 90), whereas the region in‐between with unknown function had a much lower score (mostly less than 50). However, the point mutations causing the new disease entity both affect a short alpha‐helix at the end of this region, predicted with a considerably higher confidence value (mostly above 70). Effects of a steric difference elicited by the isoleucine to asparagine or serine to proline substitution (Figure 5C) on the alpha‐helix and the function of IRF6 should be determined in future studies. Kousa and Schutte [8] speculated that underrepresentation of missense mutations along with less conservation suggests that most coding changes in *IRF6* exons 5 and 6 are either innocuous or associated with a different pathogenic process. Our findings support an association of missense mutations in exon 6 with a different disease entity. It is therefore quite possible that missense mutations actually occur with a similar frequency in exons 5 and 6 as in the other exons of *IRF6*, although these have not yet been described, since amino acid substitutions in the corresponding region of the protein do not lead to CL/CP and therefore not to VWS1 and PPS. A homozygous null mutation was shown to be lethal in mice [19] and seems to be incompatible with life also in humans, since to our knowledge no such change has been reported yet in any human patient. The only viable mouse model is a conditional knockout of IRF6 [20]. VWS1 and PPS are both autosomal dominant diseases, whereby haploinsufficiency of IRF6 leads to disruption of orofacial development. The discrepancy in phenotypic presentation between PPS and VWS1, specifically the more pronounced manifestations in the skin and genitalia, may be attributed to a dominant negative effect resulting from aberrant DNA‐binding. Most notably the missense mutation Arg84Cys in the DNA‐binding domain, that was found in many unrelated families [5], is associated with PPS (Figure 5A,B). The absence of impaired palatal development in our patients or in the two additional patients described by others [13] permits the conclusion that variants p.Ile218Asn, p.Ser220Pro and p.Ile218_Ser220delinsSerSerGlyAla do not limit the function and binding of the mutant protein to DNA, at least in the cells of the fusing palatal shelves. However, as only five different patients with specific amino acid changes in IRF6 have been identified so far, it cannot be ruled out with certainty that CL/CP could develop in other patients with similar mutations, due to the variability in the CL/CP phenotype. Studies of *IRF6* expression in humans, mice and zebrafish showed that the highest IRF6 levels can be detected in the fusing palatal shelves, hair follicles, palatal rugae, tooth germs, thyroglossal duct, external genitalia and skin [5, 21, 22]. This expression pattern indicates that IRF6 has additional functions beyond those in palate development, possibly contributing to tooth formation and differentiation of the skin and its appendages, such as the sweat glands. The results of our own immunohistochemical studies also imply that IRF6 plays an important role in the formation of the skin and/or sweat glands. Not only the expression pattern supports this assumption, but also in vivo and in vitro studies on model organisms [23, 24]. Mice with a dental epithelium‐specific Irf6 conditional knockout were shown to develop hypodontia, occasionally supernumerary incisors and molars as well as crown and root patterning anomalies [25]. Similar symptoms were observed in the human patients described so far. In our initial report [6], we considered natal teeth as a very characteristic feature of the new disease entity. Their absence in subject P3 might be explained by her genetic mosaicism [26] or by the assumption that the parents do not remember teeth that fell out shortly after birth. However, the two Asian patients, P4 and P5, also did not show natal teeth (Dr. Zhimiao Lin, personal communication), indicating that they are not as typical as initially thought.

Bilateral hearing impairment, oligodontia, anhidrosis and hidradenitis suppurativa‐like skin abscesses seem to be cardinal features of the new syndrome, and most likely the increased IRF6 activity causes the phenotype. Little is known about the function of IRF6 in otogenesis or hearing. In zebrafish models, IRF6 was shown to be expressed in the otic placode (14‐somite state) [27]. The pathogenic mechanisms underlying sensorineural deafness in our patients will be investigated in further projects.

Cell culture experiments confirmed the involvement of IRF6 in the differentiation of human keratinocytes [28]. In addition, IRF6 may be directly implicated in the development of sweat glands and tooth epithelial invagination in the mouse [29, 30], notwithstanding clear differences between sweat gland and dental papilla formation [31]. It therefore seems likely that hidradenitis suppurativa‐like symptoms, poikiloderma, anhidrosis and oligodontia are associated with IRF6. This is important to know also for clinicians, because the inability to perspire can be life‐threatening from the hour of birth [32, 33, 34, 35], not only in hot environments, but also when practicing sports [36] or during a febrile virus infection. As we found no evidence of typical clinical features of VWS1 or PPS, the amino acid residues Ile218 and Ser220 of IRF6 may be particularly relevant for epidermal cell differentiation. Recent tissue‐culture experiments of Zhang et al. [13] suggest a key role for residues 216–222 in nucleocytoplasmic trafficking and a gain‐of‐function phenotype for mutant IRF6 in which isoleucine at position 218 is replaced by asparagine or serine. However, they only investigated a single amino acid position. Their conclusion about the possible function of that domain does not explain the underrepresentation of missense variants throughout IRF6 exons 5 and 6 [8].

An important posttranslational target of IRF6 is tumour protein p63 which drives transcription of *IRF6*, while IRF6 marks p63 for proteasomal degradation [8, 37], forming a negative feedback loop. This allows keratinocytes to exit the cell cycle, thereby limiting their ability to proliferate. Pathogenic variants of either *TP63* or *IRF6* may disrupt the regulatory loop, affecting the proliferation‐differentiation balance of keratinocytes that is essential both for palate fusion and skin differentiation [38]. A direct genetic link between IRF6 and p63 was demonstrated in mice, when compound heterozygous *p63+/−* and *Irf6+/R84C* mice developed a cleft palate [39]. One could speculate that the bilateral hearing impairment observed in our patients is directly or indirectly related to p63. Hearing loss has been reported for 17% of subjects carrying pathogenic *TP63* variants [28]. In mice, TAp63, which acts via the Notch signalling pathway, is crucial for the development of the organ of Corti, providing a molecular explanation for sensorineural deafness in patients with *TP63* mutations [40]. We also believe that another cardinal feature of the new syndrome, the recurrent skin abscesses mimicking hidradenitis suppurativa, are a secondary effect of the altered IRF6‐p63 regulatory loop.

In addition, a plakophilin 1 (PKP1)‐related disease entity overlaps with conditions associated with IRF6 or p63: Doolan et al. [41] demonstrated that pathogenic *PKP1* variants not only lead to skin fragility and blistering, but also, in some cases, to hypohidrosis, pruritus, perianal erosions and dysplastic dentition. Since p63 acts as a transcription factor for *IRF6* and *PKP1* and both IRF6 and PKP1 are phosphorylated by the receptor‐interacting serine/threonine kinase 4 (RIPK4), there may also be overlaps at the molecular level.

Nevertheless, the precise role of IRF6 remains an interesting question. In particular, the actual function of the protein region encoded by exons 5 and 6, be it interaction with other proteins or dimerization, subcellular localization, improvement of protein stability or post‐translational modification [42, 43, 44, 45], is still unclear, although an important point was made by Zhang et al. [13] and further research will certainly provide additional insights.

## 5 Conclusion

A disturbed proliferation‐differentiation balance of epidermal cells due to pathogenic *IRF6* variants does not necessarily affect palatogenesis, but may cause an ectodermal dysplasia syndrome characterized by missing or dysplastic skin appendages, recurrent abscesses, poikiloderma, oligodontia and sensorineural hearing impairment. The delineation of this new syndrome facilitates a correct and early diagnosis in affected individuals and a better understanding of the IRF6‐p63 regulatory loop, which may also have implications for future therapeutic approaches to the new disease entity.

## Author Contributions

Holm Schneider, writing – original draft, acquisition and analysis of data, critical revision; Smail Hadj‐Rabia, acquisition and analysis of data; Carola Berking, data curation and critical revision; Matthias Weider, acquisition and analysis of data; Julie Steffann, acquisition and analysis of data; Ralf J. Rieker, acquisition and analysis of data; Lina Gölz, data curation and critical revision; Nicolai Peschel, acquisition and analysis of data, critical revision. All authors have read and approved the final manuscript.

## Funding

This work was funded by the German‐Austrian ectodermal dysplasia patient organization.

## Ethics Statement

The study was approved by the ethics committee of Friedrich‐Alexander‐Universität Erlangen‐Nürnberg (reference number 559_20B). All participants provided written informed consent.

## Consent

The article includes facial photographs of a patient which were taken in early infancy. The patient who is now 19 years old gave written permission to publish these photographs of himself.

## Conflicts of Interest

The authors declare no conflicts of interest.

## Data Availability

The data that support the findings of this study are available from the corresponding author upon reasonable request.
